# α-Klotho's effects on mineral homeostasis are fibroblast growth factor-23 dependent

**DOI:** 10.1097/MNH.0000000000000415

**Published:** 2015-03-15

**Authors:** Reinhold G. Erben

**Affiliations:** University of Veterinary Medicine Vienna, Vienna, Austria

**Keywords:** acute renal failure, chronic renal failure, fibroblast growth factor-23, klotho, mineral homeostasis

## Abstract

**Purpose of review:**

α-Klotho (Klotho) occurs in three isoforms, a membrane-bound form acting as a coreceptor for fibroblast growth factor-23 (FGF23) signalling, a shed soluble form consisting of Klotho's large ectodomain thought to act as an enzyme or a hormone, and a secreted truncated form generated by alternative splicing of the *Klotho* mRNA with unknown function. The purpose of this review is to highlight the recent advances in our understanding of Klotho's function in mineral homeostasis.

**Recent findings:**

A number of seminal discoveries have recently been made in this area, shifting existing paradigms. The crystal structure of the ternary FGF receptor (FGFR)-1c/Klotho/FGF23 complex has been uncovered, revealing how the ligand FGF23 interacts with FGFR1c and the coreceptor Klotho at atomic resolution. Furthermore, it was shown that soluble Klotho lacks any glycosidase activity and serves as a bona fide coreceptor for FGF23 signalling. Experiments with a combination of *Klotho* and *Fgf23*-deficient mouse models demonstrated that all isoforms of Klotho lack any physiologically relevant, FGF23-independent functions in mineral homeostasis or ageing. Finally, it was demonstrated that the alternatively spliced *Klotho* mRNA is degraded and is not translated into a secreted Klotho protein isoform in humans.

**Summary:**

Taken together, there is now overwhelming evidence that the main physiological function of transmembrane and soluble Klotho for mineral homeostasis is their role as coreceptors mediating FGF23 actions. In light of these findings, the main pathophysiological consequence of the downregulation of Klotho observed in acute and chronic renal failure may be the induction of renal FGF23 resistance.

## INTRODUCTION

Kuro-o *et al*. [1] discovered α-Klotho (Klotho) in transgenic mice in which the transgene cassette had serendipitously disrupted the function of this previously unknown gene. Homozygous *Klotho* hypomorph mice were characterized by severe growth retardation, reduced life span, vascular and soft tissue calcifications, reduced bone mass and atrophy of various organs [1]. On the basis of the phenotype of *Klotho* hypomorph mice, Klotho was thought to suppress the ageing process [1]. Klotho is a single pass transmembrane protein with a small intracellular and a large extracellular domain. The extracellular part of the protein consists of the two tandem domains KL1 and KL2, which share homology with family 1 glycosidases [1]. There are three isoforms of the Klotho protein, the full-length transmembrane form, a shed soluble form (sKlotho) produced by cleavage of the extracellular part of the protein through membrane-anchored proteolytic enzymes such as ADAM17 and a truncated soluble form produced by alternative splicing of *Klotho* mRNA [2]. The truncated Klotho protein isoform lacks the KL2 domain in mice and man [3,4], whereas the shed form consists of KL1 and KL2 but lacks the transmembrane and intracellular parts of the protein.

Klotho is mainly expressed in the kidney in proximal and distal tubules, in the choroid plexus in the brain and in the chief cells of the parathyroid gland [1,5,6]. The kidney is the major source of sKlotho in the blood, because kidney-specific ablation of *Klotho* leads to profoundly reduced circulating sKlotho levels [7]. Similarly, unilateral nephrectomy in humans reduces circulating sKlotho levels [8], corroborating the mouse data. Until recently, there has been considerable controversy about the functional role of the different Klotho isoforms. The transmembrane form of Klotho was identified as an obligatory coreceptor for fibroblast growth factor 23 (FGF23) [9]. However, the function of sKlotho has been less clear, and different explanatory models of its putative FGF23-independent mode of action have been proposed, ranging from an enzymatic function as a glycosidase to hormonal functions controlling insulin signalling in insulin target tissues, parathyroid hormone (PTH) secretion in the parathyroid gland or calcium signalling in the heart [2].

**FIGURE 1:**
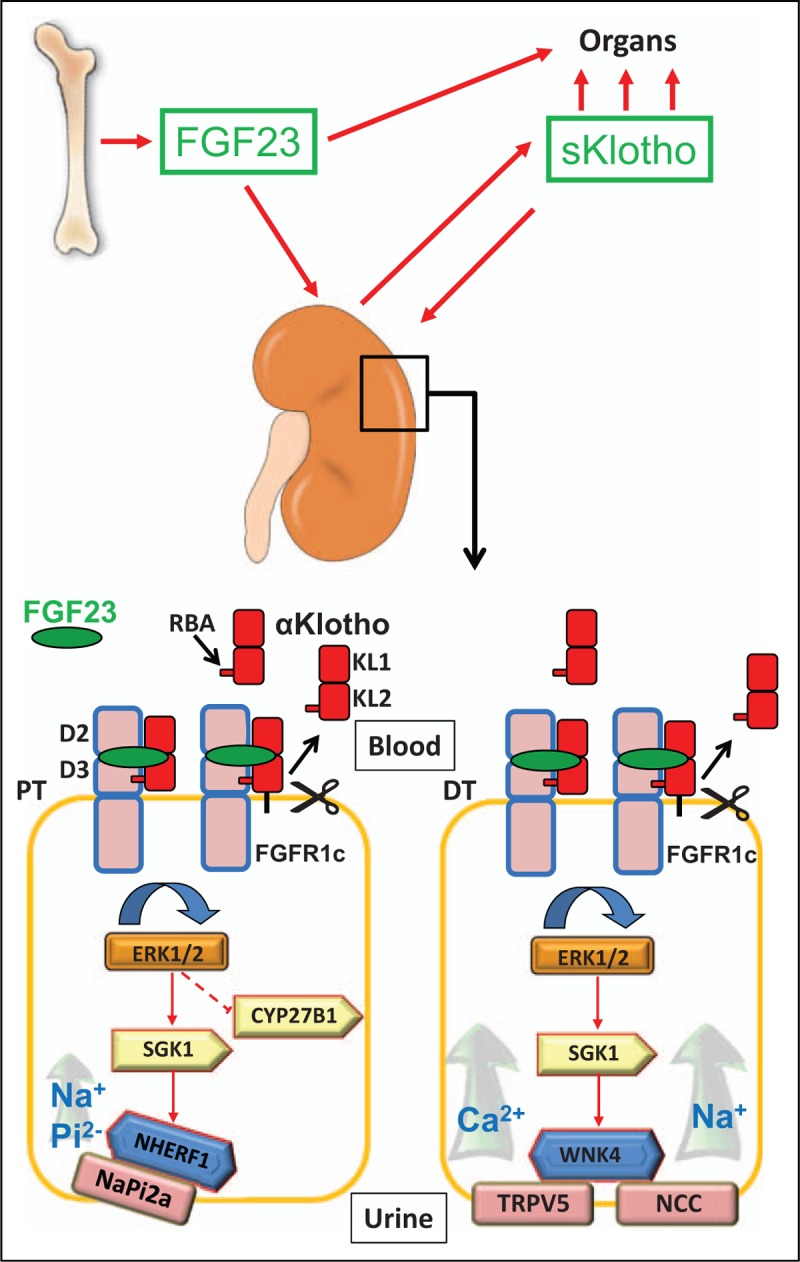
Transmembrane and soluble Klotho's role in mineral metabolism as a facilitator of FGF23 signalling in renal epithelium. FGF23 is a hormone secreted from bone and acts on the kidney and other organs. The kidney is the major source of circulating soluble Klotho (sKlotho), which is generated by shedding of Klotho's large extracellular ectodomain via membrane-anchored proteolytic enzymes. sKlotho facilitates FGF23 signalling not only in the kidney but also in other organs. Blood-borne FGF23 binds in a groove between the D2/D3 and KL1/KL2 domains of FGF receptor (FGFR)-1c and Klotho, respectively. The complex between FGFR1c, Klotho and FGF23 is stabilized by the receptor binding arm (RBA) in transmembrane and soluble Klotho. In proximal and distal renal tubules, FGF23 signalling is facilitated by either transmembrane Klotho or sKlotho circulating in the blood. In proximal renal tubular epithelium, FGF23 signalling results in activation of extracellular signal-regulated kinase 1 and 2 (ERK1/2) and of serum/glucocorticoid-regulated kinase-1 (SGK1), which in turn leads to NHERF-1 (Na^+^/H^+^ exchange regulatory cofactor-1) phosphorylation. NHERF1 phosphorylation causes degradation of membrane-bound sodium phosphate cotransporter type 2a (NaPi-2a), and a subsequent downregulation of phosphate (Pi) uptake. FGF23 also inhibits the proximal tubular expression of the 1α-hydroxylase CYP27B1, the rate-limiting enzyme for 1,25(OH)_2_D synthesis, via an unknown signalling mechanism downstream of ERK1/2. In distal renal tubules, FGF23 signalling leads to activation of with-no-lysine kinase-4 (WNK4) via ERK1/2 and SGK1 activation. WNK4 activation increases the membrane abundance of the epithelial calcium channel TRPV5 and of the sodium-chloride cotransporter NCC, leading to increased calcium (Ca^2+^) and sodium (Na^+^) uptake in the distal nephron. DT, distal renal tubule; PT, proximal renal tubule.

FGF23 belongs to the group of endocrine FGFs, which includes FGF19, FGF21 and FGF23 [10]. FGF23 is a bone-derived hormone mainly produced in osteoblasts and osteocytes. The secretion of FGF23 is stimulated by phosphate, the vitamin D hormone 1α,25-dihydroxyvitamin D_3_ [1,25(OH)_2_D], PTH, iron deficiency and pro-inflammatory cytokines [11]. Only the intact form of the protein is biologically active. The most important physiological functions of FGF23 are in the kidney, wherein it suppresses phosphate reabsorption and synthesis of 1,25(OH)_2_D in proximal renal tubules [5,12–15], and enhances calcium and sodium reabsorption in distal renal tubules [16,17]. In proximal renal tubules, FGF23 signalling downregulates the membrane expression of the sodium phosphate cotransporters type 2a and 2c (NaPi-2a and 2c) [5,14,15], whereas in distal renal tubules, FGF23 upregulates the apical membrane expression of the epithelial calcium channel transient receptor potential vannilloid-5 (TRPV5) and of the sodium-chloride cotransporter NCC [16,17] (Fig. 1). In bone, FGF23 is a potent suppressor of tissue nonspecific alkaline phosphatase, thereby regulating bone mineralization [18,19]. All paracrine and endocrine FGFs signal through the ubiquitously expressed FGF receptors (FGFRs). There are four different FGFRs, FGFR1, 2, 3 and 4, which are all tyrosine kinase receptors [20]. In addition, tissue-specific splicing leads to ‘b’ and ‘c’ variants of FGFR1, 2 and 3 [20]. Signalling of endocrine FGFs requires the concomitant presence of FGFRs and of the coreceptors α and β-Klotho [10]. The renal actions of FGF23 are Klotho dependent under physiological conditions, with FGFR1c probably being the most important FGFR mediating the hormonal actions of FGF23 [9,21].

A hallmark of *Klotho*-deficient mouse models is a premature ageing-like phenotype [1]. *Klotho*-deficient mice are characterized by early death, soft tissue calcifications, organ atrophy, osteomalacia, hypercalcemia, hyperphosphatemia and elevated circulating 1,25(OH)_2_D [1,22]. *Fgf23*-deficient mice have an almost identical phenotype [23,24]. It is now firmly established that the severe phenotype in *Klotho* and *Fgf23*-deficient mice is caused by unleashed production of 1,25(OH)_2_D due to ablation of FGF23 signalling in the kidney. In the absence of the suppressive effect of FGF23 signalling on renal 1α–hydroxylase, the normally tight regulation of this enzyme fails, and leads to excessive, unregulated production of 1,25(OH)_2_D. The subsequent intoxication with 1,25(OH)_2_D causes hypercalcemia and hyperphosphatemia, which in turn leads to soft tissue calcifications and early lethality. Consequently, ablation of the vitamin D signalling pathway rescues both *Klotho* and *Fgf23*-deficient mice [25,26]. Hence, the ageing-like phenotype in *Klotho* and *Fgf23*-deficient mice is actually caused by a disturbance of mineral homeostasis.

The purpose of this review is to highlight the recent progress in our understanding of Klotho's role in mineral metabolism. The last year has seen striking advances in this area, which collectively represent a quantum leap in our understanding of Klotho biology. 

**Box 1:**
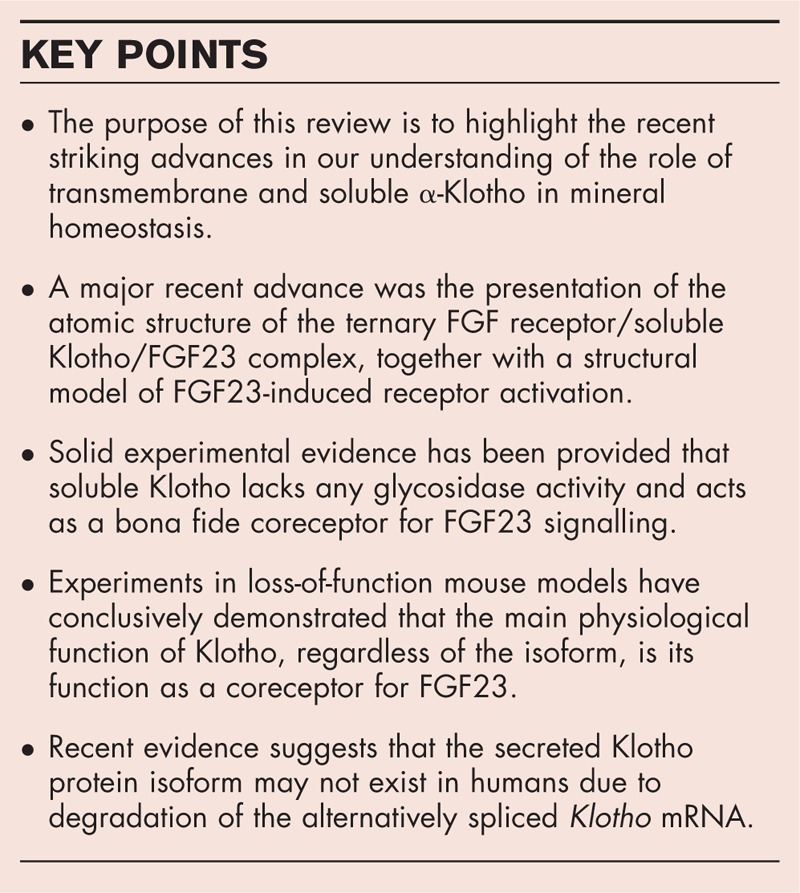
no caption available

## STRUCTURAL BASIS OF THE FIBROBLAST GROWTH FACTOR RECEPTOR-KLOTHO-FGF23 INTERACTION

Urakawa *et al*. [9] had shown that transmembrane αKlotho is an obligatory coreceptor for high affinity binding of the ligand FGF23 to FGFR1c on target cells. Transmembrane Klotho increases the binding affinity of FGFR1c to FGF23 by a factor of nearly 20 [27,28]. However, the complete atomic structure of the ligand-receptor-coreceptor complex was not known. In a recent milestone article, Chen *et al*. [29^▪▪^] crystallized the ternary complex consisting of the extracellular domain of FGFR1c, FGF23 and the Klotho ectodomain, and provided the atomic structure of the complex at 3 Ǻ resolution. In this complex, FGF23 binds in a groove formed between the D2 and D3 domains of FGFR1c, and between the KL1 and KL2 domains of Klotho, respectively. The ternary complex is stabilized by the interaction of Klotho's receptor binding arm with FGFR1c (Fig. 1). Moreover, Chen *et al.*[29^▪▪^] showed that two ternary FGFR1c/Klotho/FGF23 complexes dimerize with the help of heparan sulfate to form a 2 : 2:2 : 2 quaternary dimer complex for signal transduction. The elucidation of the 3D atomic structure of the FGFR/Klotho/FGF23 complex is a major achievement, because it significantly improves our understanding of the molecular mechanisms involved in FGF23 signalling. This improved knowledge may form the basis for the future development of small molecule modulators of this signalling pathway.

## MODE OF ACTION OF SOLUBLE KLOTHO

During the past two decades, there has been a considerable controversy regarding the role of soluble Klotho in the regulation of mineral metabolism and organ function. It has been proposed that sKlotho may act, in a FGF23-independent manner, as a hormone inhibiting insulin signalling [30], suppressing PTH secretion in the parathyroid gland [31], and protecting the myocardium by inhibiting calcium signalling through binding to ganglioside-containing lipid rafts [32,33]. However, all attempts to characterize a specific receptor for sKlotho, apart from FGFRs, have failed [2]. Alternative models of sKlothos's function in mineral metabolism are built on an enzymatic activity of sKlotho as glucuronidase or sialidase [34–37], thereby modulating the function and abundance of membrane glycoproteins by changing their glycosylation patterns.

As mentioned above, the KL1 and KL2 domains of Klotho are homologous to family 1 glycosidases. However, both domains lack one of the two essential active site glutamates, which are highly conserved in this family of glycosidases [34]. Nevertheless, it has been proposed that sKlotho regulates renal calcium, potassium and phosphate handling through Klotho's putative enzymatic activity in a FGF23-independent manner. Chang *et al*. [35] and later Cha *et al*. [37] reported that Klotho regulates calcium metabolism by changing the glycosylation pattern of the TRPV5 and TRPV6 calcium channels through its putative sialidase activity, thereby increasing the apical membrane abundance and channel activity of TRPV5 and TRPV6 in the distal nephron. A similar mechanism was proposed for the renal outer medullary potassium channel 1 (ROMK1) [38]. Furthermore, Hu *et al*. [36] reported that Klotho, through a putative glucuronidase activity, is able to suppress phosphate reabsorption from urine by inhibiting the activity of NaPi2a in renal proximal tubular epithelium.

However, the recent seminal study by Chen *et al*. [29^▪▪^] has provided solid experimental evidence that Klotho lacks any biologically relevant glycosidase activity. The work of the latter authors refutes the possibility that sKlotho regulates mineral metabolism through its enzymatic activity. Rather, Chen *et al*. [29^▪▪^] showed that soluble and transmembrane Klotho possess similar capacities to facilitate FGF23 signalling *in vitro*. Furthermore, injection of recombinant sKlotho into wild-type mice resulted in a small, but significant increase in urinary phosphate excretion. Importantly, when the latter authors injected a mutated form of sKlotho lacking the FGF receptor binding arm into normal mice, they found a striking downregulation of FGF23 target genes in the kidney, together with hyperphosphatemia. These findings indicate that the effects of sKlotho on mineral metabolism require interaction with FGFRs. Hence, mutant sKlotho lacking the FGF receptor binding arm acts as a dominant negative coreceptor *in vivo*, interfering with FGF23 signalling in target tissues. In summary, Chen *et al*. [29^▪▪^] have clearly and convincingly shown that sKlotho serves as a soluble coreceptor for canonical FGF23 signalling (Fig. 1).

This notion is supported by additional, independent lines of evidence: Hum *et al*. [39^▪^] recently reported that both stable delivery of sKlotho by a viral vector, and acute injection of sKlotho lowered serum phosphate in *Klotho*-deficient mice. In addition, they observed a profound stimulation of bony FGF23 secretion in *Klotho*-deficient mice overexpressing sKlotho, an effect that could be explained by the FGFR1-dependent stimulation of osteoblastic FGF23 secretion by FGF23 and sKlotho. Similarly, an earlier study by Shalhoub *et al*. [40] also demonstrated that sKlotho enhances the suppressive effect of FGF23 on alkaline phosphatase in mouse osteoblast-like cells in an FGFR1-dependent manner. Collectively, these findings are in full agreement with the notion that sKlotho is an on-demand circulating coreceptor facilitating FGF23 signalling in different tissues (Fig. 1).

Current open questions in this context are: First, what is the relative contribution of transmembrane Klotho vs. sKlotho–mediated FGF23 signalling in target tissues under normal circumstances? Second, is shedding of sKlotho in the kidney regulated? Third, as FGFR1c is expressed almost ubiquitously, does sKlotho enhance FGF23 signalling in a body-wide fashion? In principle, biologically relevant levels of circulating sKlotho should render all target cells expressing FGFR1c FGF23-sensitive. In an attempt to answer the question regarding the relative contribution of transmembrane vs. soluble Klotho for renal FGF23 signalling, we recently cotreated 200 μm thick live kidney slices isolated from wild-type and *Klotho*-deficient mice *ex vivo* with sKlotho and FGF23 [41^▪▪^]. We found that cotreatment with sKlotho did not modulate the FGF23-induced changes in renal tubular calcium, sodium or phosphate transport monitored by 2-photon microscopy in wild-type and *Klotho*-deficient kidney slices, suggesting that transmembrane Klotho is by far more important than sKlotho for FGF23-mediated signalling in the kidney under near-physiological circumstances [41^▪▪^]. However, a caveat of the latter model is that the results may be biased by different migration velocities of proteins with grossly different sizes such as sKlotho (130 kDa) and FGF23 (32 kDa) in intact tissues. Therefore, it is unclear whether the latter model is predictive of the in-vivo situation.

Collectively, the abovementioned studies have provided solid evidence that neither transmembrane nor soluble Klotho have FGF23-independent functions. Chen *et al*. [29^▪▪^] conclude that ‘In light of these data, we propose that the pleiotropic antiaging effects of α–Klotho are all dependent on FGF23’. A recent study performed in our laboratory fully corroborates this conclusion. To examine the existence of physiologically relevant, FGF23-independent effects of Klotho on mineral homeostasis *in vivo*, we generated triple knockout mice with simultaneous deficiency in *Fgf23* and *Klotho* and a nonfunctioning vitamin D receptor (VDR) (*Fgf23*^−/−^*/Klotho*^−/−^/VDR^▵/▵^*, Fgf23/Klotho*/VDR). The rationale behind crossing *Fgf23* and *Klotho*-deficient mice with VDR mutant mice is that *Fgf23* and *Klotho* null mice can be studied at older ages only when vitamin D signalling is ablated [25,26]. We found that *Fgf23/Klotho*/VDR triple knockout mice were phenocopies of *Fgf23/*VDR double knockout mice, and that *Fgf23/Klotho* double knockout mice were phenocopies of single *Fgf23* knockout mice, independent of age or sex [41^▪▪^]. These results strongly argue against any physiologically relevant, FGF23-independent effects of Klotho, regardless of its isoform, on mineral homeostasis or ageing. Taken together, there is now overwhelming evidence that the main physiological function of transmembrane and soluble Klotho for mineral homeostasis *in vivo* is their role as coreceptors mediating FGF23 actions (Fig. 1).

## SECRETED FORM OF KLOTHO

The third isoform of Klotho is a truncated form produced by alternative splicing. It was reported that alternative splicing of the *Klotho* mRNA gives rise to a secreted, truncated Klotho protein isoform in mice and humans [3,4], but not in rats [42]. Murine alternatively spliced *Klotho* mRNA lacks exons 4 and 5 [4], whereas a premature stop codon leads to truncation of the Klotho protein in man [3]. Hence, both the murine and the human secreted Klotho protein isoforms consist of KL1 only and lack the KL2 and transmembrane domains. The lack of the transmembrane domain led to the assumption that the alternative mRNA transcripts code for a secreted isoform of Klotho [3,4].

Notably, the paradigm-shifting study by Mencke *et al*. [43^▪▪^] reported that the premature stop codon responsible for putative truncation of the Klotho protein in humans primes the alternatively spliced mRNA for degradation. Hence, these recent data suggest that there may be no secreted Klotho protein in man. Whether a secreted Klotho protein isoform exists in mice has never been robustly tested. Moreover, the data provided by Chen *et al.*[29^▪▪^] indirectly suggest that even if a secreted Klotho protein exists, it should be functionally inactive. First, neither KL1 nor KL2 possess biologically relevant glycosidase activity. Therefore, secreted Klotho cannot serve as an enzyme. Second, as both tandem domains are required for the interaction between the ligand FGF23 and Klotho [29^▪▪^], it can be inferred that the isolated KL1 domain is unable to act as a coreceptor. Hence, the recent advances in the field have cast serious doubts on the notion that a secreted form of Klotho serves any biological function.

## PATHOPHYSIOLOGICAL ROLE OF KLOTHO IN ACUTE AND CHRONIC RENAL FAILURE

The advances in Klotho biology described above may have major implications for the pathophysiology of renal disease. There is good evidence from clinical and experimental studies that both acute and chronic renal failure are associated with reduced expression of Klotho in the kidney, and with reduced sKlotho levels in the blood [43^▪▪^,^44^–^47^]. In agreement with these data, kidney transplantation in CKD patients results in a rapid rise in circulating sKlotho, and a decline in circulating FGF23 levels [48^▪^,49^▪^]. Conversely, treatment of mice with sKlotho improves outcome in acute and chronic renal injury models [50^▪▪^]. In light of the above-mentioned novel data regarding the role of sKlotho as a facilitator of FGF23 signalling, we propose that the main pathophysiological effect of the decreases in renal Klotho expression and circulating sKlotho in acute and chronic renal failure may be the induction of renal FGF23 resistance. Thus, the protective effects of sKlotho in experimental renal failure models [46,50^▪▪^] may at least partially be explained by a reduction of renal FGF23 resistance, a subsequent reduction of serum FGF23 levels as shown recently by Hu *et al*. [50^▪▪^], and, hence, indirect protection against FGF23-induced renal and cardiovascular damage. In this context, the recent finding by Smith *et al*. [51^▪▪^] that FGF23 is a profibrotic factor in the kidney by stimulating injury-primed fibroblasts may help to explain why an FGF23-lowering effect of sKlotho may *per se* have antifibrotic effects.

## CONCLUSION

Recently, major advances have been made in the field of Klotho's role in mineral metabolism. First, the atomic structure of the FGFR1c/sKlotho/FGF23 complex, together with a structural model of FGF23-induced receptor activation, has been revealed. Second, it has been shown that soluble Klotho is an on-demand coreceptor for FGF23 signalling. Third, it was demonstrated that the main physiological function of Klotho, independent of its isoform, is its function as a coreceptor for FGF23. Fourth, solid evidence has been provided showing that the alternatively spliced *Klotho* mRNA is degraded and is not translated into a secreted Klotho protein isoform in humans. Collectively, these advances represent a major step in our understanding of Klotho biology and may have important implications for the pathophysiology of acute and chronic renal failure.

## Acknowledgements

None.

### Financial support and sponsorship

This work was supported by a grant from the Austrian Science Fund (FWF P24186-B21) to R.G.E.

### Conflicts of interest

The author declares no conflicts of interest.

## REFERENCES AND RECOMMENDED READING

Papers of particular interest, published within the annual period of review, have been highlighted as:

▪ of special interest
▪▪ of outstanding interest
